# Hemispheric Specialization Varies with EEG Brain Resting States and Phase of Menstrual Cycle

**DOI:** 10.1371/journal.pone.0063196

**Published:** 2013-04-30

**Authors:** Stephanie Cacioppo, Francesco Bianchi-Demicheli, Paul Bischof, Dominique DeZiegler, Christoph M. Michel, Theodor Landis

**Affiliations:** 1 Department of Psychology, High Performance Electrical NeuroImaging (HPEN) Laboratory, Center for Cognitive and Social Neuroscience (CCSN), The University of Chicago, Chicago, Illinois, United States of America; 2 Department of Gynecology and Obstetrics, University Hospital and Medical Faculty of Geneva, Geneva, Switzerland; 3 Department of Obstetrics and Gynecology II, Reproductive Endocrine and Infertility, Hôpital Cochin-St Vincent de Paul, Paris, France; 4 Department of Neurology, University Hospital and Medical Faculty of Geneva, Geneva, Switzerland; Ecole Normale Supérieure, France

## Abstract

A growing body of behavioral studies has demonstrated that women’s hemispheric specialization varies as a function of their menstrual cycle, with hemispheric specialization enhanced during their menstruation period. Our recent high-density electroencephalogram (EEG) study with lateralized emotional versus neutral words extended these behavioral results by showing that hemispheric specialization in men, but not in women under birth-control, depends upon specific EEG resting brain states at stimulus arrival, suggesting that hemispheric specialization may be pre-determined at the moment of the stimulus onset. To investigate whether EEG brain resting state for hemispheric specialization could vary as a function of the menstrual phase, we tested 12 right-handed healthy women over different phases of their menstrual cycle combining high-density EEG recordings and the same lateralized lexical decision paradigm with emotional versus neutral words. Results showed the presence of specific EEG resting brain states, associated with hemispheric specialization for emotional words, at the moment of the stimulus onset during the menstruation period only. These results suggest that the pre-stimulus EEG pattern influencing hemispheric specialization is modulated by the hormonal state.

## Introduction

Using high-density electroencephalogram (EEG) recordings, we recently demonstrated that hemispheric specialization depends upon functional brain states (FBS) at stimulus onset for men, but not for women [1]. In Mohr et al’s study, men showed, subsequent to one specific FBS-class, a strong emotional word advantage after left visual field-right hemisphere (LVF-RH) presentation, and none after right visual field-left hemisphere (RVF-LH) presentation [1]. On the other hand, the same participants showed a moderate emotional word advantage for both visual field subsequent to another specific FBS-class. This suggests that two fundamentally different reading strategies can occur in the same individual as a function of the momentary functional brain states (FBS): one without and one with a strong hemispheric specialization [1].

FBSs can be defined electrically by means of the momentary configuration of the global scalp electric potential, reflecting the momentary neuronal cerebral activity. Based on previous studies indicating that topographic differences at stimulus arrival directly indicate that different neuronal populations are active, one may assume that different distributions of active neuronal populations serve different functions, and thus represent different momentary functional states of the brain [1 for review]. Accordingly, a FBS corresponds to an EEG brain topography that is stable over time, and that is sustained by a specific neural network. In previous work, Lehmann and collaborators have classified these FBS that occurred at stimulus arrival, and calculated separate evoked-potentials for each one of them [2]–[4]. The authors then showed that stimulus-evoked potentials drastically varied as a function of such different classes of FBS, independent of task complexity. These findings, thus, suggest that subtle variations of FBS may influence subsequent event-related information processing.

From an electrical neuroimaging viewpoint, it has been shown that the momentary electrical “brain state” at stimulus onset (FBS) might be the crucial determinant of the fate of subsequent information processing as FBSs precede a stimulus onset [5], [6]. In Mohr et al.’s study, this suggests that specific FBS-classes, occurring at stimulus arrival, are characteristic of functional hemispheric specialization, while other types of FBS-classes are not [1].

From a functional neuroimaging viewpoint, the relation between endogenous sex steroid levels and functional brain connections remains still poorly understood as functional cerebral asymmetries vary not only as a function of the period of the menstrual cycle and sex hormones but also as a function of the cognitive task used. Discrepancies among the neuroimaging results of functional cerebral asymmetries also exist as a consequence of different methodologies and paradigms. For instance, one study found a general increase in brain activation with increasing estradiol levels [7], but no changes in functional cerebral asymmetries between the menstrual and the follicular phase. Another fMRI study showed the extent of language-related activations to be significantly correlated with progesterone and less strongly with estradiol levels [8]. Yet another study investigated the influence of estradiol on the change of functional cerebral asymmetries across the menstrual cycle by means of a connectivity analysis in 14 normally cycling women, who performed a word-matching task, while they were scanned twice during the cycle, once during the menstrual and once during the follicular phase [9]. They showed that inhibitory influences of left-hemispheric language areas on homotopic areas of the right hemisphere was strongest during the menses (cycle day 1–3), resulting in a pronounced lateralization, while during the follicular phase (cycle day 9–11), with rising estradiol levels functional cerebral asymmetries were reduced [9].

Based on this literature, one could hypothesize that women who are not under birth control could present specific FBS-classes that are characteristic to hemispheric specialization during some (but not all) of the phases of their menstrual cycle. This question hasn’t been addressed, yet. In Mohr et al.’s study, women had artificially regulated hormonal cycle (i.e., women under birth control) and didn’t show any FBS-class specific to hemispheric lateralization [1].

The goal of the present study was thus to show whether the FBS at stimulus onset would be related to hemispheric specialization at some phases of the menstrual cycle.

## Materials and Methods

### Participants

A total of 22 women responded to the call for participants and volunteered to participate in our lexical decision study that was posted via flyers. Twelve of the volunteers responded to our inclusion criteria, and were thus selected to do the EEG part of our study. [Exclusion criteria were then the following: 1) the use of oral contraceptives within the previous six months; 2) menstrual cycles that were longer than 30 days or shorter than 26 days or that were irregular (i.e., abnormal variation in length); 3) current treatment with anabolic steroids or psychoactive medications; 4) or a history of neurological or psychiatric disorders.] We obtained informed written consent from all twelve participants involved in our study, which was approved by the Ethical Committee of the University Hospital of Geneva, Switzerland.

All participants were right-handed (Edinburg Inventory; [10]), and had normal or corrected-to-normal visual acuity. None had any prior or current neurological or psychiatric impairment, as ascertained by a detailed medical history and the Hospital Anxiety and Depression scale. Prior to the cognitive experimental sessions, every participant was seen by a gynecologist (DDZ), a psychiatrist (FBD) and a neuropsychologist (SC), in order to assure normal menstrual and neuropsychological functioning.

### Procedure

#### Control of participants’ hormonal levels

During the study, an experimental protocol has been set up to assure that measurements occurred at the same hormonal periods in every participant. To assess the ovulating date of every participant’s menstrual cycle, an ovarian ultra-sound was performed by an experienced gynecologist 12 days after the first day of the participant’s menstruation period in order to determine with precision the follicular period (i.e., follicule >13 mm). Follicule-Stimulating hormone, Luteinizing hormone, estradiol and progesterone levels were assessed to evaluate the menstrual cycle phases (See Table S1). Table S1 confirms that the participants’ hormonal levels were in the normal range for every period. For instance, the early luteal phase was characterized by hormonal levels that significantly differed from the hormonal levels collected from the other phases of the menstrual cycle (estradiol, Follicule-Stimulating Hormone; *p*<.05; Progesterone; *p*<.01). As expected, participants had also significantly high Luteinizing Hormone levels during the early luteal phase.

### Procedure

To avoid any biases in the recording phase of the menstrual cycle, every woman’s brain activity and behavioral performance was recorded during each one of the five phases of their menstrual cycle (once during the menstrual phase (cycle day 3), once during the late follicular period (day 12), once during the early luteal phase (ovulation period detected from LH surges in urine for each participant), once during the mid-luteal phase (day LH+7), and once during the late-luteal period (LH+11)). The order of testing was balanced across the cycle. Directly after the session corresponding to the ovulation period (LH), the LH+7 and LH+11, a blood sample was collected. LH, FSH, estradiol and progesterone levels are assessed to evaluate the menstrual cycle phases in women. Blood analyses were performed as described above at the Department of Hormonology at the University Hospital of Geneva, Switzerland.

During each phase, participants completed five blocks, each containing 120 trials. As such, each subject’s participation required approximately 40 minutes, including time for electrode cap application and clean up. Before inclusion in this study, potential subjects completed a training session comprised of a shortened version of the five blocks of stimuli described above, but with different word and non-word stimuli. Inclusion in the experiment required a minimum of 50% accuracy on each condition. The beginning of the recording session was counterbalanced across subject as a function of their menstrual cycle period. Some participants started experimental testing during their menstruation phase and others began testing during their early luteal phase.

### Stimuli

The stimuli consisted of 112 letter-strings (four-to-seven characters long; [11]). As in Ortigue et al., 2004 [11], these 112 letter-strings included: i) eight emotional French abstract nouns (e.g., colère (anger), amour (love)), i) eight French neutral abstract nouns (e.g., chose (thing), ligne (line), File S1), iii) 48 pronounceable non-words following the same consonant-vowel structure as words, and iv) and another set of 48 pronounceable non-words (each non-word was created according to the same consonant–vowel structure as its associated contro-laterally presented non-word constituting 24 non-word/non-word pairs). The first 48 non-words were used to create word/non-word pairs. Since words were repeated three times per block, a different non-word was presented for each word/non-word pair.

These words come from a set of 215 neutral and emotional French words with high frequency of usage. The emotional connotation of the words was ascertained by thirty volunteers, comprised of nurses and medical doctors from the University of Geneva Hospital, who did not participate in the EEG experiment, and rated each word’s emotional content on a scale from 0 to 7 (0 = unemotional; 7 = very emotional). The eight words with the highest and lowest ratings were used in the present EEG experiment (see [11] for further details). The selected emotional and neutral words had a mean usage frequency of 186 and 226 occurrences per million words, respectively. No significant difference in word frequency was observed between the emotional and non-emotional words [*t*(7) = 0.14; *p* = .89; paired t test].

### Experimental Paradigm

Letter-strings were presented in pairs – one on either side of central fixation (spanning ∼2°–5° eccentricity) in the left visual field and right visual field, respectively, in a go/no-go paradigm. This resulted in four experimental conditions: emotional words presented in the right or left visual field (hereafter Emotional Right Visual Field and Emotional Left Visual Field, respectively) and neutral words presented in the Right or left visual field (hereafter Neutral Right Visual Field and Neutral Left Visual Field, respectively).

More precisely, words, when present, were always paired with non-words and could appear randomly on either side of fixation, but with equal overall likelihood across the experiment. Each word appeared three times in each visual field per block of trials. The order of experimental trials was pseudorandom. No more than three consecutive trials with the same word type appeared in the same visual field. Stimuli appeared white on black for 13 ms, which was confirmed by photocell measurements (E-prime Psychology Software Tools Inc., Pittsburgh, USA) on a computer monitor located 140 cm from the subject, whose head position was stabilized in a chin rest. The inter-stimulus interval varied randomly from 1500 to 2000 ms.

### Participants’ Instruction

Participants were instructed to decide whether one of the two letter strings was a word or not. They had to centrally fixate and judge whether or not a word was present and, if so, on which side it appeared (Figure S1). That is, if they believed a word was presented, they pressed a button as quickly as possible with their index finger of the hand on the same side of the fixation cross as the word (go-trials).

As in previous studies [e.g., [11]], participants were not explicitly asked to make a decision on the emotional nature of the words. Catch trials involved presentation of two non-words and required no button-press. However, as described above, for analysis purposes, conditions containing words were further and equally subdivided into those containing emotional or neutral words.

### Electrophysiological Data Acquisition & Analysis

During the behavioral bilateral lexical decision task, continuous electroencephalogram (EEG) was recorded from 128 AgCl carbon-fiber coated electrodes using an Electric Geodesic Sensor Net® (GSN200; Electrical Geodesic Inc., Oregon). The EEG was digitized at 500 Hz, band-width filtered at 0.01–200 Hz, with the vertex electrode (Cz) serving as an on-line recording reference; and impedances were kept below 50 kΩ. Data were offline recalculated to the average reference and filtered between 1–30 Hz.

### Determination of Functional Brain States at Stimulus Onset

The momentary global functional state of the brain is determined by the distribution of all active neuronal populations at one given moment in time. The sum of these neuronal activations can be measured on the scalp surface as an EEG potential map with certain topography. It has been shown in numerous studies that the configuration of these maps remain stable for a certain amount of time (∼100 ms) and then change rapidly in a new configuration in which they stay stable again. These periods of stable map topography have been termed functional microstates. During a microstate, only the electric strength of the field varies, not the topography. A global measure of field strength is the global field power, which corresponds to the spatial standard deviation of the potential field. The global field power maxima are thus the best representatives of a given microstate in terms of signal-to-noise ratio. As in previous studies on state-dependent information processing, we determined the scalp potential map at the global field power peak in a time window between 100 ms before to 10 ms after stimulus onset as representing the momentary functional brain state at the moment of stimulus presentation.

The map at the timeframe of maximum global field power in this 110 ms temporal window was extracted and concatenated for all participants. Due to noise in the electrophysiological data caused by oculomotor artifacts, EEG data from two participants had to be rejected. Thus the results described here after correspond to the electrophysiological analyses of ten participants.

All participants were concurrently submitted to a hierarchical agglomerative cluster analysis in order to identify the most dominant scalp topographies across all subjects during two phases of their menstrual cycle: the menstruation period (baseline) and the early luteal phase. The early luteal phase was chosen as an exemplar period of the menstrual cycle to compare it with baseline menstruation period because this early luteal phase is characterized by hormonal levels that are significantly different from the other phases and a behavioral emotional advantage that differs from baseline.

During this cluster analysis, the polarity of the maps was ignored, and a modified Krzanowski-Lai criterion was applied to define the optimal number of clusters (or “template maps”). The Krzanovski-Lai criterion searches for the L-corner of one of the quality measure of the clustering procedure, the dispersion curve. This means that the optimal number of clusters is selected when additional clusters do not lead to a significantly increased global quality (for review see [12]–[14]).

The global template maps resulting from this cluster analysis were grouped in two classes according to their spatial similarity (one with a Left Anterior–Right Posterior orientation of the positive and negative maxima (LA-RP maps) and another class with a Right Anterior–Left Posterior orientation (RA-LP maps), as we did in the study of Mohr et al. [1], and the mean maps of these two classes were determined. These two map classes were then back fitted into the original data of the participants by using a competitive fitting procedure. This procedure allows to calculate the spatial correlation between each original map and the two template maps and to assign the template map with the higher correlation to each original map [13]. This fitting procedure allowed to determine the total number of times each template map was observed in each condition of each subject.

### Data Analyses

In order to elucidate whether lexical decision performance was dependent on the FBS maps, we calculated individuals’ percent of correct responses for each condition. First, we summed for each individual, all artifact-free and correct trials for each functional brain state class separately. This sum score was used for each functional brain state class as individuals’ reference to calculate percent correct responses for each word type in each visual field (i.e., Left and Right visual field). Then, as in previous studies, we calculated an emotional word advantage index score for each one of the FBS topographies separately for each visual field [Emotional Word Advantage index = ((Emotional- Neutral)/(Emotional+Neutral))*100]. According to this laterality index score, POSITIVE values indicate an emotional word advantage and NEGATIVE values a neutral word advantage.

### Statistical Analyses

These emotional word advantage index scores were subjected to an analysis of variance (ANOVA). Post-hoc simple comparisons were also performed. Results were accepted as significant *at p*<.05.

## Results

### Behavioral Results

#### Reaction times

Analysis of the reaction times revealed a significant main effect of word class (*F*(1, 45) = 63.94; *p*<.0001; emotional<neutral: *M_emotional_* = 542.89 ms (SD: 13.66)<*M_neutral_* = 586.39 (SD: 15.34)) and visual field (*F*(1, 45) = 116.92; *p*<.0001; RVF<LVF: *M_RVF_ = *515.60 ms (SD: 12.39)<*M_LVF_ = *613.69 ms (SD: 17.92)), indicating that participants were faster at identifying emotional words and words appearing in the right visual field (RVF) than neutral words and words presented to their left visual field (LVF). The interaction between these two factors also reached significance (*F*(1, 45) = 9.50, *p* = .004). In line with previous studies (e.g., [15]), post-hoc comparisons also showed a significant difference between emotional and neutral words when presented to LVF (*F*(1, 45) = 63.16, *p*<.0001). No significant effect of the menstrual cycle was observed across the five phases (*F*(4, 45) = 0.10, *p* = .98) and the interaction between the three factors (word class, visual field and phase of the menstrual cycle) did not reach significance either (*F*(4, 45) = 2.12, *p* = .09).

#### Accuracy

Analysis of accuracy revealed a significant main effect of word class (*F*(1, 45) = 71.78; *p*<.0001; emotional>neutral: *M_emotional_ = *73.16% (SD: 2.65)<*M_neutral_ = *60.94% (SD: 3.49)) and visual field (*F*(1, 45) = 102.20; *p*<.0001; *RVF*>*LVF*: *M_RVF_ = *78.2% (SD: 2.53)>*M_LVF_ = *55.9% (SD: 3.95)). The interaction between these two factors also reached significance (*F*(1, 45) = 26.77, *p*<.0001), suggesting that participants detected better emotional words than neutral words, especially when presented in the RVF. No significant effect of menstrual cycle was observed across the five different phases (*F*(4, 45) = 0.17, *p* = .95). There was also no significant interaction between the three factors (*F*(4, 45) = 0.13, *p* = .97).

In line with the reaction times and previous studies, post-hoc comparisons showed a significant difference between emotional and neutral words when presented to the LVF (*F*(1, 45) = 75.77, *p*<.0001). Interestingly, post-hoc simple comparisons of each phase of the menstrual cycle also revealed that emotional word advantage was significantly greater in LVF than RVF during the menstruation phase (*F*(1, 9) = 7.8611, *p* = .02), marginally significant during the follicular phase (F(1, 9) = 5.8, *p* = .04) and not significant during the early, mid- and late luteal phases (*p*>.05).

#### Emotional Word advantage

**Figure 1:**
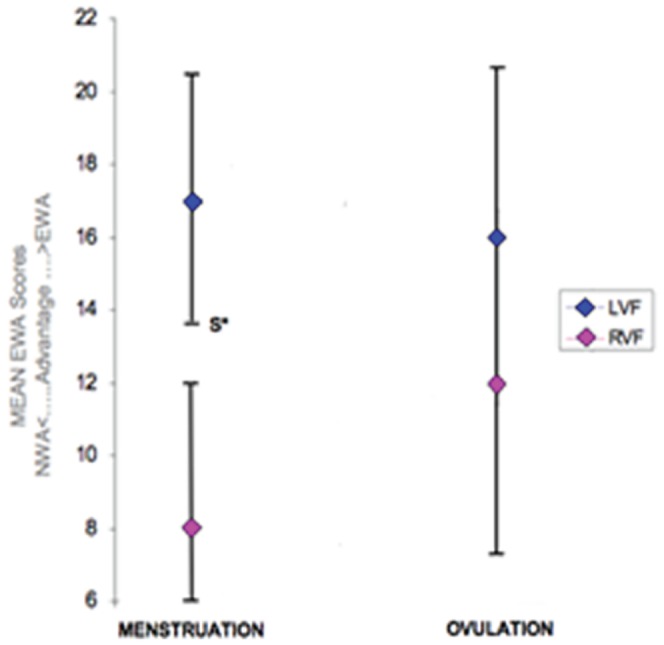
Mean emotional word advantage (EWA) index scores for correct lexical decisions after word presentation to the right visual field (RVF) and left visual field (LVF). Positive values indicate an emotional word advantage and negative values a neutral word (NWA) advantage. Index scores are displayed for two phases of the menstrual cycle (MENSTRUATION; and EARLY LUTEAL PHASE). Asterisks indicate significant group differences and vertical bars indicate standard errors. *p values <.05.

**Figure 2:**
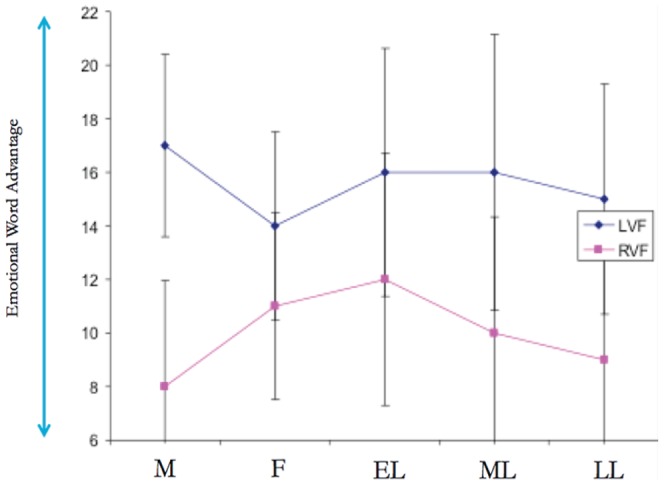
Mean emotional word advantage (EWA) index scores for correct lexical decisions after word presentation to the right visual field (RVF) and left visual field (LVF) across the different phases of the menstrual cycle.

As in Mohr et al.’s study [1], a behavioral emotional word advantage (EWA) index score was calculated for each condition in order to facilitate the comparison between the two studies. Behavioral results are presented in Figure 1 (See also Figure 2 for further details). Analysis of the emotional word advantage and microstates is described below.

### Microstate Results

**Figure 3:**
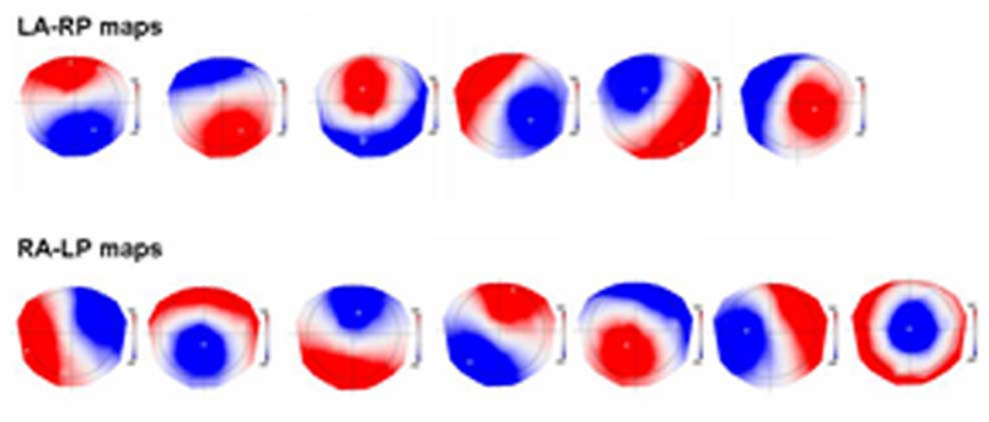
The 13 maps determined from the spatial cluster analysis. Shown in the upper row are the six functional brain state maps with a left anterior-right posterior (LA-RP) orientation of the extrema and in the lower row those with a right anterior-left posterior (RA-LP) orientation.

From the xy maps that entered the cluster analysis (subjects x conditions x all artifact free sweeps), the Krzanowski-Lai criterion identified 13 different maps that best described the data (Figure 3). As can be seen from the figure, like in the previous study of Mohr et al., [1] two distinct classes of map topographies are seen in these 13 maps: one with a Left Anterior–Right Posterior orientation of the positive and negative maxima (LA-RP maps) and another class with a Right Anterior–Left Posterior orientation (RA-LP maps). As shown in Figure 1, six out of the 13 maps had a similar LA-RP orientation (upper row, Figure 3), and seven maps had a similar RA-LP orientation (lower row; Figure 3). As in Mohr et al., [1] we assured that these two map classes were different by calculating the median spatial correlation within and between the classes. This analysis revealed a median correlation of 0.73 for the maps in the LA-RP class and a median correlation of 0.60 for the maps in the RA-LP class, while the median correlation between the maps of the two classes was only 0.39. After this grouping into the two map classes we calculated the mean map of each class and determined the number of times these two mean maps were present in the two conditions and the different stimulus types.

Mean percentage (+/− SD) of presence of these two FBS classes (Left Anterior-Right Posterior and Right Anterior-Left Posterior) across all artifact-free epochs for correct lexical decision and across participants are shown in Table 1 (for MENSTRUATION) and Table 2 (for EARLY LUTEAL PHASE). Values are presented for emotional and neutral words in the left visual field and right visual field for the two phases across participants.

**Table 1:** Mean percentage +/− SD number of frequencies for each FBS class (LA-RP and RA-LP) across all artifact-free epochs for correct lexical decision as well as across the 10 participants during the MENSTRUATION phase.

MENSTRUATION			
	LA-RP maps	RA-LP maps	
ELVF	53^+/−7.2^	47^+/−7.2^	
NLVF	42^+/−9.7^	58^+/−9.7^	
ERVF	52^+/−6.3^	47^+/−6.3^	
NRVF	48^+/−7.5^	52^+/−7.5^	

Values are presented for emotional (E) and neutral (N) words in the left visual field (LVF) and right visual field (RVF) for the MENSTRUATION phase.

**Table 2:** Mean percentage +/− SD number of frequencies for each FBS class (LA-RP and RA-LP) across all artifact-free epochs for correct lexical decision as well as across the 10 participants during the EARLY LUTEAL phase.

EARLY LUTEAL PHASE		
	LA-RP maps	RA-LP maps
ELVF	42^+/−12.4^	58^+/−12.4^
NLVF	47^+/−18^	53^+/−18^
ERVF	45^+/−11.4^	55^+/−11.4^
NRVF	46^+/−15^	54^+/−15^

Values are presented for emotional (E) and neutral (N) words in the left visual field (LVF) and right visual field (RVF) for the EARLY LUTEAL phase across all 10 participants.

**Figure 4:**
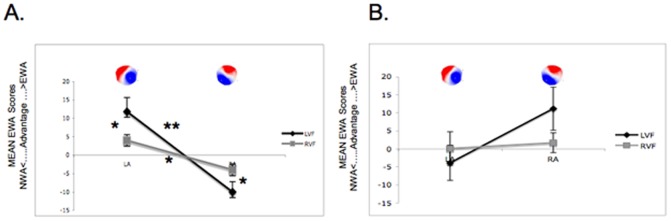
Functional brain state classes as a function of the period of the menstrual cycle. Index scores are displayed for two phases of the menstrual cycle (MENSTRUATION, A; and EARLY LUTEAL PHASE, B) and two classes of FBS (LA-RP, the so-called LA, and RA-LP, the so-called RA) separately. Positive values indicate an emotional word advantage (EWA) and negative values a neutral word (NWA) advantage. Asterisks indicate significant group differences and vertical bars indicate standard errors. *p values <.05, **p values <.01.

Figure 4AB displays FBS classes for each visual field and emotional word advantage index scores across MENSTRUATION and EARLY LUTEAL phases (see also Table 3). Statistical analysis on this emotional word advantage index score showed a significant three-way interaction between phase of the menstrual cycle, visual field and functional brain state orientation [*F*(1,9) = 9.17, *p* = .01], suggesting that the more women are in a Left Anterior-Right Posterior oriented functional brain state at the time of word presentation to their left visual field-right hemisphere during the MENSTRUATION phase, the stronger the emotional word advantage is (Figure 4A).

**Table 3:** EWA index scores for each visual field and across the MENSTRUATION and EARLY LUTEAL phases.

	MENSTRUATION		EARLY LUTEAL PHASE	
Index score	LA-RP	RA-LP	LA-RP	RA-LP
LVF	11.8	−10.2	−3.87	6.4
RVF	4.3	−4.04	0.08	1.7

This index score reflects an emotional (E) over neutral (N) advantage for each visual field separately [Emotional Word Advantage (EWA) index = (E-N)/(E+N)*100; (79)]. According to this index score, POSITIVE values indicate an EWA and NEGATIVE values a neutral word advantage (NWA).

In support of these findings, post-hoc analyses were performed for the MENSTRUATION phase (see also Figure 4A) and showed a significant difference between right and left visual fields for Left Anterior-Right Posterior maps (paired t-test *t*(9) = 2.13; *p* = .03; Figure 4A), suggesting that Left Anterior-Right Posterior are more present for emotional word advantage in the left visual field during MENSTRUATION phase.

Interestingly, the opposite dissociation was observed for Right Anterior-Left Posterior maps, suggesting that Right Anterior-Left Posterior are more present for neutral word advantage in the left visual field. A significant difference was also observed between left and right visual field for Right Anterior-Left Posterior maps (*p* = .02). Furthermore, a significant difference was observed between Left Anterior-Right Posterior and Right Anterior-Left Posterior, suggesting that Left Anterior-Right Posterior was more dominant within the left visual field (*t*(9) = 3.33; *p*<.01). Similarly, within the right visual field, a significant difference was observed between Left Anterior-Right Posterior and Right Anterior-Left Posterior (*t*(9) = 2.66; *p* = .01).

Post-hoc analyses for the EARLY LUTEAL phase were also performed (see Figure 4B). No significant difference was observed between left and right visual field for Left Anterior-Right Posterior maps (*t*(9) = 1.1; *p* = .15) or for Right Anterior-Left Posterior maps (*t*(9) = 1.72; *p* = .06). Similarly, no significant difference was found between Left Anterior-Right Posterior and Right Anterior-Left Posterior for the right visual field (*t*(9) = 0.22; *p* = .4) or the left visual field (*t*(9) = 1.4; *p* = .09).

## Discussion

The present findings demonstrate a specific Left Anterior-Right Posterior oriented functional brain state at stimulus arrival during the MENSTRUATION phase compared to the EARLY LUTEAL PHASE period. More precisely, we found a significant interaction of the emotional word advantage index score between phase of the menstrual cycle, visual field and functional brain state orientation, suggesting that the more participants are in a Left Anterior-Right Posterior oriented functional brain state at the time of word presentation to their left visual field-right hemisphere during the MENSTRUATION phase, the stronger the emotional word advantage is. Hemispheric specialization pattern subsequent to the Left Anterior-Right Posterior class during MENSTRUATION is strongly enhanced, showing a significant emotional word advantage for the left visual field-right hemisphere and the reverse, a significant “neutral word advantage” after presentation to the right visual field-left hemisphere, while during the EARLY LUTEAL phase subsequent to the Left Anterior-Right Posterior class no significant effect is found, indicating “bi-hemispheric” treatment. Our results suggest that these differences occur because “laterality-enhancing” functional brain state’s (Left Anterior-Right Posterior oriented maps) are more frequently present during MENSTRUATION than during EARLY LUTEAL PHASE.

In line with Hausmann and Güntürkün’s hypothesis, which postulates a “progesterone-mediated inter-hemispheric decoupling” [16]–[18], Hausmann and collaborators assumed that steroid hormones, especially progesterone, reduce functional cerebral asymmetries, presumably through their glutamatergic and GABAergic effects that diminish cortico-cortical transmission [16]–[18]. Recent studies have shown that other hormones could also influence functional cerebral asymmetries [9], [16], [18]–[21]. Despite a growing body of evidence about the role of sex hormones on functional cerebral asymmetries [22], [23], the mechanisms by which sex hormones operate on brain lateralization remain unclear. Endogenous levels of estradiol and progesterone vary considerably during the menstrual cycle with low levels of both estradiol and progesterone during menses, and high levels of estradiol during the follicular phase of the cycle (i.e. just before ovulation around day 10) with the highest level being reached immediately before the luteinizing hormone surge (ovulation, 14 days), and progesterone reaching its peak in the mid-luteal phase around day 22 [24], [25]. After ovulation, there is a decline in the estradiol level, which then gradually increases during the early luteal phase (approximately 15 to 19 days), and the maximum rates of post-ovulatory estradiol secretion are attained during the advanced luteal phase (approximately 20 to 25 days) [24], [25]. In the premenstrual phase (approximately 26 to 28 days), the level of estradiol secretion decreases. The progesterone levels remain low during the follicular phase. Shortly after ovulation, the level of progesterone secretion increases steadily, peaks during the mid-luteal phase, and declines precipitously thereafter [24]. As a function of the different levels of steroid hormone concentration during the menstrual cycle, behavioral studies show inconsistent lateralization effects as a function. For instance, some studies found largest asymmetries during cycle phases high in steroid hormone concentration [26], [27], while other studies found maximal asymmetries during the low steroid menses [22], [28]. Critical behavioral differences have been also demonstrated between menstruation periods and other phases of the menstrual cycle, with women in their menstruation period having similar lateralized performances than men. That said, other hormonal levels, such as FSH and LH are higher in the early luteal phase compared to other phases, and thus could play a role in the modulation of hemispheric lateralization.

In terms of microstate analysis, four dominant maps are typically identified in the ongoing multichannel resting EEG that show highly anti-correlated temporal behavior, i.e. only one of them is present during a certain time period of about 100 ms [29]–[31]). Two of these four maps closely resemble the two maps identified in the presented study, i.e. a RA-LP map and a LA-RP map. In a combined EEG-fMRI study Britz and Michel [30] could show that each of the dominant microstate maps is correlated with one typical fMRI resting state network. The RA-LP map correlated with the auditory-phonological resting state network, and the LA-RP map with the visual resting state networks described in Mantini et al.’s study [32]. Interestingly an earlier study by Lehmann et al. [33] attributed the same functions to these two maps, long before fMRI resting state networks were described. In their study, they classified spontaneously reported daydreams into visual and abstract thoughts and then looked at differences between the spontaneous EEG maps during these two classes of thoughts. They found that the LA-RP map was more present when subjects had rather visual daydreams, and the RA-LP map when they had more abstract (verbal) thoughts. Given these previous results on spontaneous EEG, one could speculate that when subjects are in the “visual state” (the LA-RP map) before stimulus onset, they rather treat the stimuli visually, while words are treated in a more abstract verbal fashion when subjects are in the “abstract state” (the RA-LP maps) before stimulus arrival. This might explain the stronger and lateralized emotional word advantage during the MENSTRUATION phase, where the women showed increased presence of the visual LA-RP map, and the more bilateral treatment of the words in the EARLY LUTEAL phase when the abstract-verbal brain state dominates.

A limitation of the present study is that it included only a small number of participants. Replication with a greater number of subjects will increase our confidence in the generalizability of these findings, and help to better understand the specific relationship between microstates and functional cerebral asymmetries, as a function of sex hormonal modulations and electric brain dynamics.

## Supporting Information

Figure S1 Experimental paradigm. Letter-string were presented bilaterally as participants centrally fixated.
(EPS)

File S1 List of French words used during the present lexical decision task.
(DOCX)

Table S1 Mean and standard deviations for hormone levels during menstrual cycle phases.
(DOC)
