# Supplementary material for: Hemispheric Specialization Varies with EEG Brain Resting States and Phase of Menstrual Cycle
Source: PLoS One. 2013 Apr 30;8(4):e63196. doi: 10.1371/journal.pone.0063196 (PMC3640095; doi:10.1371/journal.pone.0063196)
Supplement: File S1 — List of French words used during the present lexical decision task. (DOCX) [file pone.0063196.s002.docx]

**File S1.** **List of French words used during the present lexical decision task.**

**Neutral Words**

**actuel (actual)**

**bout (end)**

**cause (cause)**

**chose (thing)**

**fait (fact)**

**ligne (line)**

**sorte (sort)**

**truc (trick)**

**Emotional words**

**colère (anger)**

**espoir (hope)**

**joie (joy)**

**mort (death)**

**plaisir (pleasure)**

**rêve (dream)**

**sexe (sex)**

**viol (rape)**
