# Supplementary material for: Hemispheric Specialization Varies with EEG Brain Resting States and Phase of Menstrual Cycle
Source: PLoS One. 2013 Apr 30;8(4):e63196. doi: 10.1371/journal.pone.0063196 (PMC3640095; doi:10.1371/journal.pone.0063196)
Supplement: Table S1 — Mean and standard deviations for hormone levels during menstrual cycle phases. (DOC) [file pone.0063196.s003.doc]

**Table S1. Mean and standard deviations for hormone levels during menstrual cycle phases.**

Early luteal Mid Luteal Late Luteal

**Mean** ± **St Dev Mean** ± **St Dev Mean** ± **St Dev**

Estradiol 129.45±78.4*^a^ 107.75±67.67 66.99±30.37

(pg/mL)

(normal range: 40.58-428.02)^c^ (normal range: 101.01-465.95)^c^ (normal range:20.73-385.52)^c^

Progesterone 1.8±1.7*** 13.91±5.13 7.03±4.86

(ng/mL)

(normal range: 0.77-18.47)^c^ (normal range: 5.86-28.79)^c^ (normal range: 0.38-28.03)^c^

FSH 6.69±5.3*^b^  2.24±1.26 1.88±1.08

(IU/L)

(normal range: 2.22-17.04)^c^ (normal range: 0.92-7.34)^c^ (normal range: 0.89-7.67)^c^

Luteinizing hormone 16.95±12.76** 3.11±2.4 1.91±1.09

(IU/L)

(normal range: 2.03-26.17)^c^ (normal range: 0.68-12.12)^c^ (normal range: 0.21-8.02)^c^

* Significant Paired t-test: a) early vs. late luteal, *p* < .05; b) early vs. mid- and late luteal, *p* < .05.

** Significant Paired t-test: early vs. mid- luteal, *p* = .007; early vs. late luteal, *p* = .005.

*** Significant Paired t-test: early vs. mid luteal, *p* < .01; early vs. late luteal, *p* = .01.

c: Source:

Stricker R, Eberhart R, Chevailler M-C, Quinn FA, Bischof P, et al. (2006) Establishment of detailed reference values for luteinizing hormone, follicle stimulating hormone, estradiol, and progesterone during different phases of the menstrual cycle on the Abbott ARCHITECT analyzer. Clinical chemistry and laboratory medicine CCLM FESCC 44: 883–887.
